# *Thought Chart*: tracking the thought with manifold learning during emotion regulation

**DOI:** 10.1186/s40708-018-0085-y

**Published:** 2018-07-19

**Authors:** Mengqi Xing, Johnson GadElkarim, Olusola Ajilore, Ouri Wolfson, Angus Forbes, K. Luan Phan, Heide Klumpp, Alex Leow

**Affiliations:** 1 Department of Bioengineering, University of Illinois at Chicago, Chicago, IL USA; 2 Department of Computer Science, University of Illinois at Chicago, Chicago, IL USA; 3 Department of Psychiatry, University of Illinois at Chicago, Chicago, IL USA; 4 Department of Psychiatry, Department of Bioengineering, University of Illinois at Chicago, Chicago, IL USA

**Keywords:** *Thought Chart*, Nonlinear dimensionality reduction, Nearest neighbor, Manifold learning, EEG connectome, Emotion regulation

## Abstract

The Nash embedding theorem demonstrates that any compact manifold can be isometrically embedded in a Euclidean space. Assuming the complex brain states form a high-dimensional manifold in a topological space, we propose a manifold learning framework, termed *Thought Chart*, to reconstruct and visualize the manifold in a low-dimensional space. Furthermore, it serves as a data-driven approach to discover the underlying dynamics when the brain is engaged in a series of emotion and cognitive regulation tasks. EEG-based temporal dynamic functional connectomes are created based on 20 psychiatrically healthy participants’ EEG recordings during resting state and an emotion regulation task. Graph *dissimilarity space embedding* was applied to all the dynamic EEG connectomes. In order to visualize the learned manifold in a lower dimensional space, local neighborhood information is reconstructed via k-nearest neighbor-based nonlinear dimensionality reduction (NDR) and epsilon distance-based NDR. We showed that two neighborhood constructing approaches of NDR embed the manifold in a two-dimensional space, which we named *Thought Chart*. In *Thought Chart*, different task conditions represent distinct trajectories. Properties such as the distribution or average length in the 2-D space may serve as useful parameters to explore the underlying cognitive load and emotion processing during the complex task. In sum, this framework is a novel data-driven approach to the learning and visualization of underlying neurophysiological dynamics of complex functional brain data.

## Background

The Nash embedding theorems [1, 2] showed that any Riemannian *n*-manifold with a $C^{1}$ positive metric has an isometric embedding in a Euclidean space of dimension 2*n*+1, even in any small portion of this space. Since the Gaussian curvature of a surface is invariant under local isometry based on the *Theorema Egregium* [3], the manifold properties in a low-dimensional space can provide an insight into the topological structure of the brain. Recent advent in structural and functional neuroimaging has further suggested that brain imaging data may construct a smooth and differentiable manifold, in which local neighborhood properties can assist in recognizing the underlying pattern in trajectories of brain development [4], discriminating different types of brain tumors [5], and improving the imaging registration accuracy [6]. However, the manifold associated with temporally varying brain dynamics imaging data has not yet been explored. Here, we propose a framework to examine the *intrinsic geometry* of the mind’s topological space of functional brain imaging via dissimilarity-based manifold learning.

We assume brain states compose a high-dimensional space [4, 7], which can be reconstructed and visualized in a low-dimensional space via dimensionality reduction. Common dimensionality reduction approaches including principal component analysis (PCA) and linear discriminant analysis (LDA) are often used in functional brain networks to visualize select regions of interest [8, 9]. However, a linear approach cannot recognize nonlinear structures in a high-dimensional space [9], making it unsuitable for preserving the global intrinsic geometry for complex dynamic brain data. Nonlinear dimensionality reduction approach isomap yields global coordinates which provide a simple way to analyze and manipulate high-dimensional observations in terms of their intrinsic nonlinear degrees of freedom and produce a globally optimal low-dimensional Euclidean representation [10].

The functional network data are informed by electroencephalogram (EEG) for its high temporal resolution, coupled with the emotion regulation task. The synchronization of response systems is highly dynamic when human brains are engaging in emotion and cognitive tasks [11]. Thus, with proper sectioning, the synchronicity between regions of a brain network can be described, in the form of a connectome, using a phase-based connectivity analyses approach, weighted phase lag index (WPLI) [12]. Guided by our own published research results, theta wave may be the most sensitive among all EEG frequencies in investigating EEG networks in emotion regulation task (ERT) [13, 14]. Therefore, we selected theta wave network connectivity as the input for our manifold learning.

Here, we propose a manifold learning framework, *Thought Chart*, to explore the underlying intrinsic geometry of dynamic functional networks. The hypothesis of this manifold learning is that the reconstructed manifold will reflect different properties of the brain’s state during tasks. Additionally, by sampling the space, we can extract specific aspects of the trajectory in this manifold that reflect task performance, such as coordinates and their distribution and levels of scattering [15]. To recover the local neighborhood information needed for the nonlinear dimensionality reduction (NDR) step, here we tested two strategies in searching for local neighbors: K-nearest neighbor (KNN), which sets each point to search for its k-nearest points; and epsilon radius, which sets each point to search for all points within a fixed radius $\varepsilon$. Properties of *Thought Chart* constructed by these two approaches will be evaluated to see how neighborhood identification influences the intrinsic geometry of human brain.

## Methods

### Data acquisition and emotion regulation tasks (ERT)

**Fig. 1:**
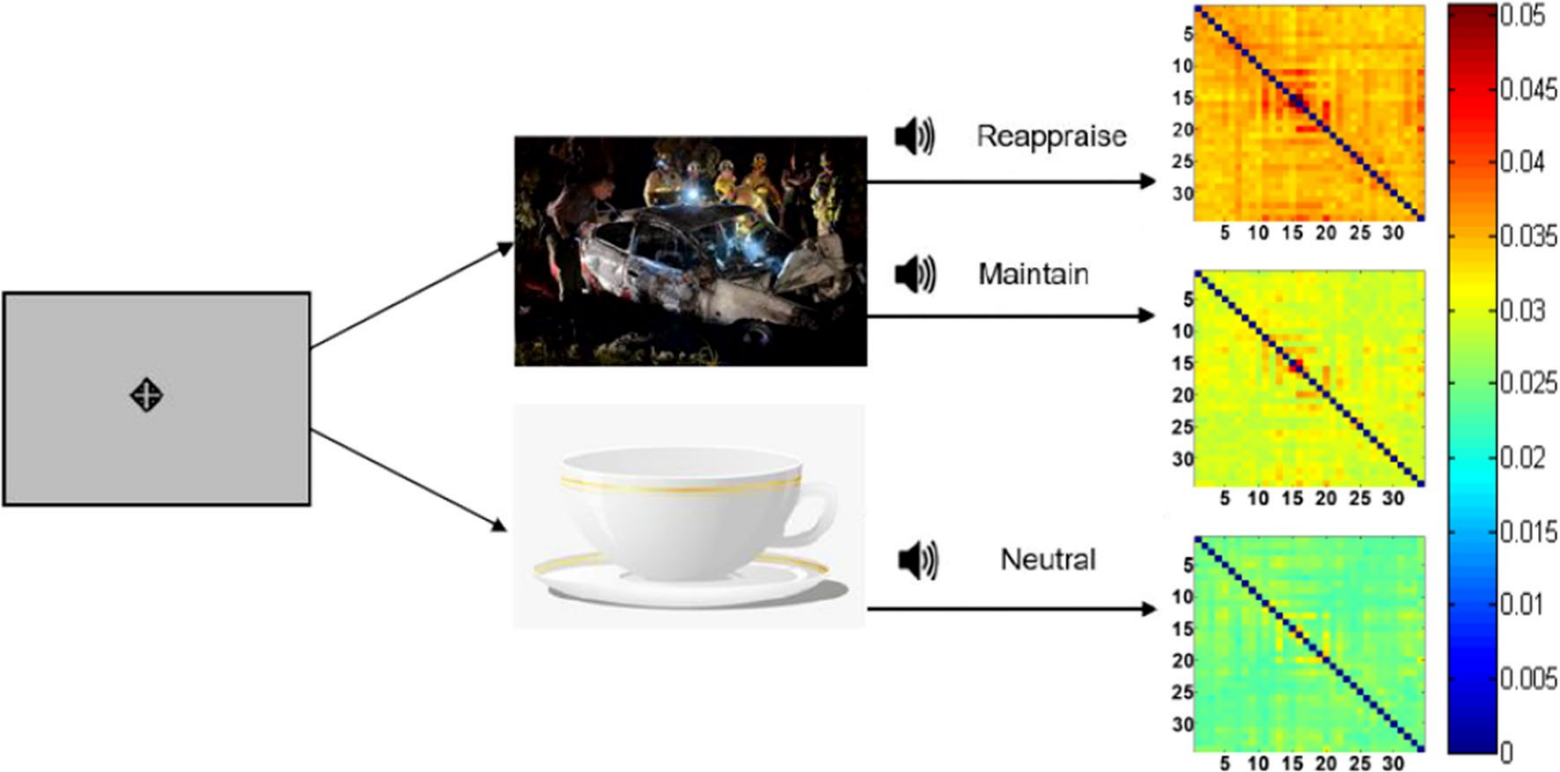
An illustration of a typical ERT session. A fixation point is on display before each trial, then followed by either a neutral or negative picture on the screen. An audio instruction will ask test subjects to *maintain*, reappraise or stay neutral

EEG data were collected from 20 psychiatrically healthy participants (age: $27.2\pm 9.3$ ) using the Biosemi system (Biosemi, Amsterdam, the Netherlands) with an elastic cap with 34 scalp channels. Each participant underwent one session of ERT (Fig. 1). During ERT, participants were requested to look at pictures displayed on the screen and listen to a corresponding auditory guide. Two types of pictures will be on display for 7 s in random orders: emotionally neutral pictures (landscape, everyday objects, etc.) and negative pictures (car crash, nature disasters, etc.). An auditory guide will come after the picture on display for 1 s, instructing the participant to “Look”: viewing the neutral pictures; to “*Maintain*: viewing the negative pictures as they normally would; or to “Reappraise”: viewing the negative pictures while attempting to reduce their negative emotion by reinterpreting the meaning of pictures [16, 17]. A separate session of eight-minute eyes-open EEG resting states was recorded for later manifold learning. All EEG data were preprocessed using Brain Vision Analyzer (Brain Products, Gilching, Germany), by first segmenting task trials into 7 s segments. A sliding window in size of 0.5 s and a step size of 0.05 were applied to create the dynamic data. (The first and last five time points were discarded, resulting in 130 time points per session.) Frequencies of interest were set from 1Hz to 50Hz in increments of 1Hz. The final output of each subject was averaged over trials within the same task. Resting-state data were processed under the same parameter. Due to the non-trial-based setting of the recording, resting state will only serve as bases that further create contrast in manifold learning. Thus, the manifold properties associated with the resting-state connectomes in the Euclidean space are not included in our final analyses.

### Weighted phase lag index-based EEG connectome

As functional communications between two brain regions result in synchronized or phase-coupled EEG readouts, in this study we used *weighted phase lag index* (WPLI) computed [12] between the times series of two channels to form EEG connectomes (each of which is a symmetric 34 by 34 matrix). Mathematically, WPLI is defined as:

1 $$\begin{aligned} \hbox {WPLI}_{xy}=\frac{n^{-1}\sum _{t=1}^{n}\left| imag(S_{xyt}) \right| sgn(imag(S_{xyt}))}{n^{-1}\sum _{t=q}^{n}\left| imag(S_{xyt}) \right| } \end{aligned}$$

where $imag(S_{xyt})$ indicate the cross-spectral density at time *t * in the complex plane *xy*, and *sgn* is the sign function $(-1, +1 or 0)$ [12]. The connectivity matrices were generated with the *MATLAB * toolbox *Fieldtrip * (Donders Centre for Cognitive Neuroimaging, Nijmegen, the Netherlands). The final output time-dependent EEG connectome for an individual task of each subject is arranged as $34 \times 34\times 50\times 130$ $(channel \times channel \times frequency \times time ).$ In this study, we primarily focused on the manifold informed by theta wave (4–7 Hz) in EEG connectomes.

### Learning the manifold with *graph dissimilarity space embedding*

To collect sufficient amount of data points to learn the intrinsic geometry of a high-dimensional manifold, we utilized the EEG connectomes from all subjects at *all* time points as sampling possible states of the manifold that is shared among all subjects. Then, graph *dissimilarity space embedding* is used to represent each connectome as a point in a very high-dimensional space *(over* $10^{4}$ *)*, which is described below [18, 19]. Assuming labeled sample graph set $G={G_{1},..., G_{n} }$ has n ”prototype” graph observations $G_{i} \in \mathbb {G}$ (the set of all possible graphs under consideration) and d is the distance metric that can be computed between two graphs $d: \mathbb {G} \times \mathbb {G} \rightarrow [0,\infty )$, then any graph $X \in \mathbb {G}$ can be represented using $\varphi _{n}^{G}:\mathbb {G} \rightarrow \mathbb {R}n$, defined as the n-dimensional vector $\varphi _{n}^{G}(X) = [d(X, G_{1}), ... d(X, G_{n})]$. In this way, any graph set can be represented by a set of real numbers. In our case, all the connectomes were initially used as prototypes for an unsupervised learning. Thus, the number of dimensions is in the same order as the number of observations in the dataset.

Reconstructing the local neighborhood reconstruction. Here, we emphasize that this step is crucial in order to properly learn the manifold’s *intrinsic geometry*, as *d* (which is used to define coordinates in the embedding space, and thus not intrinsic to the manifold) will not properly inform geodesics (the shortest paths on the manifold, which is an intrinsic property) except in local neighborhoods. While such a construction calls for a “good” choice of the distance function *d*, we posit that given a sufficiently large amount of data points the learned manifold will converge to the true manifold with any reasonably chosen *d*. Given two connectome matrices *X* and *Y*, a natural choice, which we adopted here, is the Euclidean distance: $d(X,Y)=\sqrt{\sum _{ij}(X_{ij}-Y_{ij})^{2} }$ and $\left\| \varphi _{n}^{G}(X)-\varphi _{n}^{G}(Y)\right\| =\sqrt{\sum _{k}(d(Y,G_{k})-d(X,G_{k}))^{2}}$ .

### Nonlinear dimensionality reduction

Once the local neighborhood is learned, a matrix representing this high-dimensional manifold was reconstructed into a lower dimensional Euclidean space via NDR. Once this is achieved, *Thought Chart* of any given individual can be constructed by tracing the trajectory of the time-dependent connectome of that subject for any given task. In this case, we selected isomap, a nonlinear prototypical isometric embedding procedure which entails the computation of geodesics based on neighborhood information followed by the (quasi-)isometric embedding of the geodesics.

To provide a good approximation to geodesic distance, the first step of isomap is to determine the neighbors of each point on the low-dimensional manifold based on the distance matrix acquired from the previous step [20]. Here, we compared two common approaches to determine whether two points are neighbors: the k-isomap method and the $\varepsilon -isomap$ method. K-isomap utilized the k-nearest neighbor algorithm to determine neighbors, while $\varepsilon$-isomap includes all the points within some fixed radius $\varepsilon$. The relationships of the neighborhood are represented in a weighted graph D in which $D(X, Y) = d(X, Y)$ if X and Y are neighbors, otherwise$d(X, Y) = \infty$ [20]. The second step relies on applying the *classic multidimensional scaling* (MDS) to the centered squared geodesic distance matrix, whose eigendecomposition provides the basis for lower dimensional embedding.

### Exploration of ERT *Thought Chart*

The resting state and the emotion regulation will be visualized in a two-dimensional Euclidean space. To quantify the dynamic properties and thought trajectory of emotion regulation, we summarize the Euclidean distance along the trajectory in this 2-D space (average length) and the average distance from the centroid (spreadness) for each subject. For each subject with time length *t* $\in [1,130]$ , the average trajectory length is described as:

2 $$\begin{aligned} L=\sum _{t=1}\sqrt{(x_{t}-x_{t+1})^{2}+(y_{t}-y_{t+1})^{2}}/(130-1) \end{aligned}$$

where *x*, *y* are the position of each point in the 2-D space. And the spreadness is described as:

3 $$\begin{aligned} S=\sum _{t=1}^{t=130}\sqrt{(x_{t}-\bar{x})^{2}+(y_{t}-\bar{y})^{2}}/130 \end{aligned}$$

## Results

### *Thought Chart* construction

**Fig. 2:**
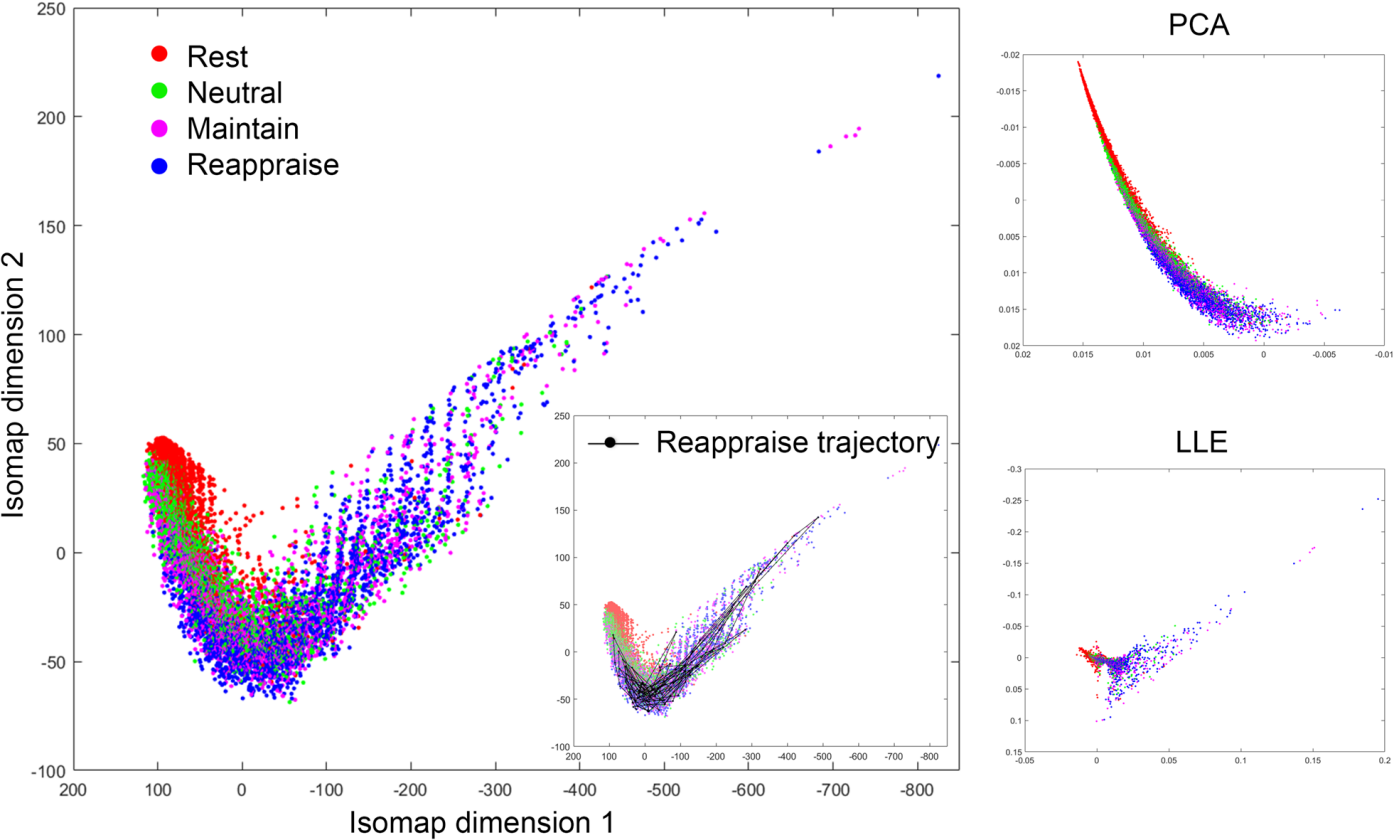
An example *Thought Chart* during Reappraise learned from the temporal EEG connectomes of 20 healthy subjects, both at rest and during ERT, using NDR methods of isomap (left) and LLE (lower right), as well as standard PCA (upper right). Visually, NDR methods yielded an ordered transition from resting, *Neutral, Maintain* to *Reappraise* along the manifold’s principal dimension (isomap dimension 1)

**Fig. 3:**
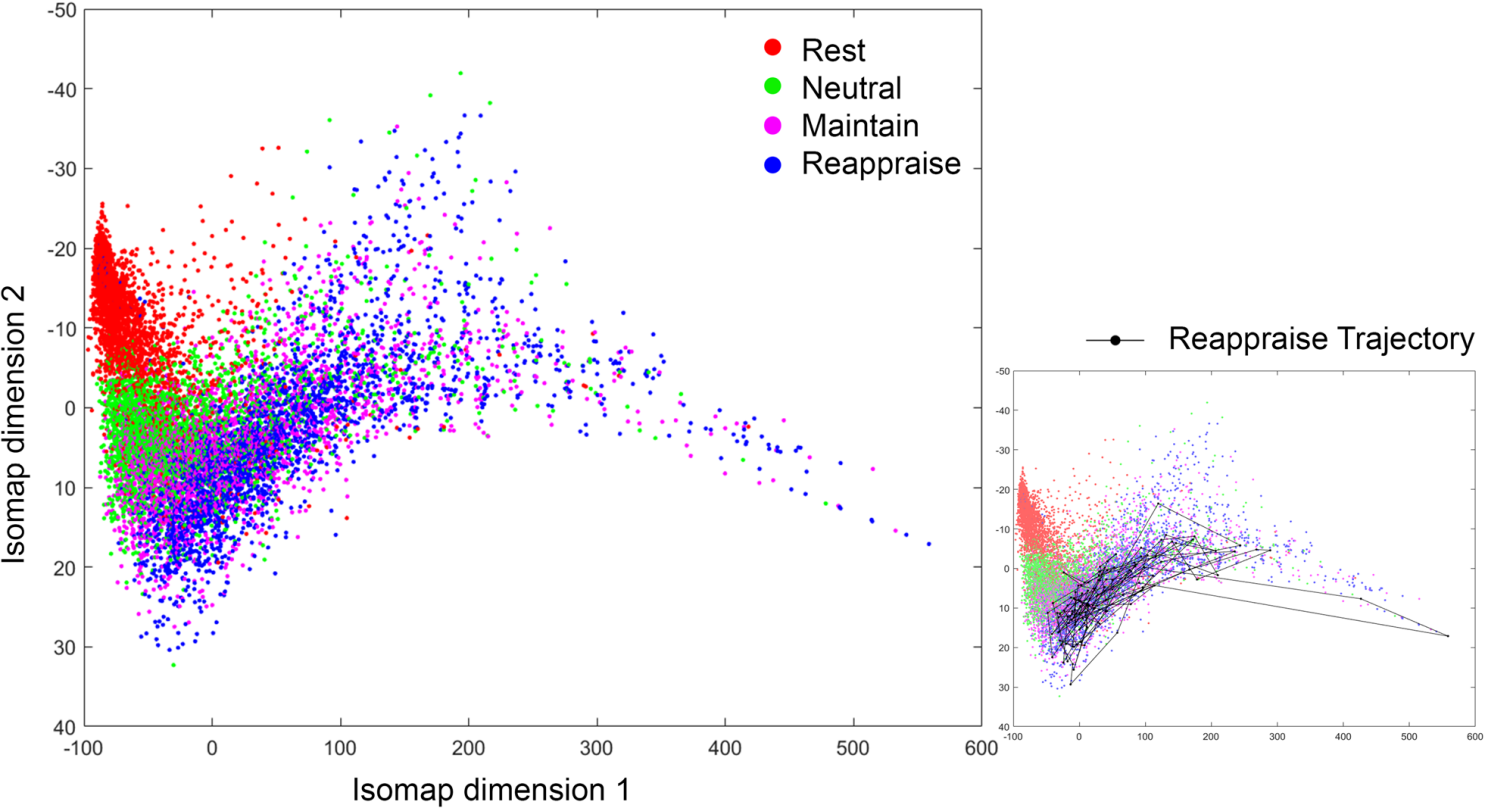
An example *Thought Chart* using NDR method of $\varepsilon$-isomap, *Thought Chart* follows the same ordered transition of resting, *Neutral*, *Maintain* to *Reappraise*

After averaging across theta frequencies (4–7 Hz) and combining both resting and ERT theta connectomes for all time points, 20 healthy subjects thus contributed a total of 10400 connectomes ($130\times 20 \times 4$). We repeated our analyses with a range of *k* and $\varepsilon$ values. In *k*-isomap, the trajectory length difference across three conditions is stable with *k* ranging from 10 to 120. Though we presented results with $k=30 \,(0.3 \%$ of the total points), the reported differences in tasks are consistent with any *k* in this range (Fig. 8a). In the case of $\varepsilon$-isomap, a point can potentially be excluded after the neighborhood construction step if it has no neighbors within an $\varepsilon$ radius (unlike in k-isomap, all nodes are retained after neighborhood construction). Therefore, the number of connected components is the main factor in selecting the $\varepsilon$. When $\varepsilon$ is larger than 28, the size of connected components converges to a constant, where 10,371 out of 10,400 points are connected (Fig. 8b). As the number of dimensions reduced from 10,400 to 2, the reconstructed theta EEG manifold exhibited a *principal* dimension that is shared by all four states in both isomaps (x-axis in Figs. 2, 3) with a secondary up–down motion from one side to the other. Visually, this manifold thus resembles the shape of a snake by spiraling around its main axis. Moreover, the distribution along the first dimension follows an ordered transition: (from low to high amplitude) resting (red), *Neutral* (green), *Maintain* (purple) and *Reappraise* (blue), corresponding to increasing cognitive load of the tasks. The similar task distribution was observed with *locally linear embedding* (LLE) [21] (Fig. 2), a non-isometric NDR approach. Additionally, the embedding generated using simple PCA (a linear technique) does not recover the full nonlinear distribution seen in either isomap or LLE.

**Fig. 4:**
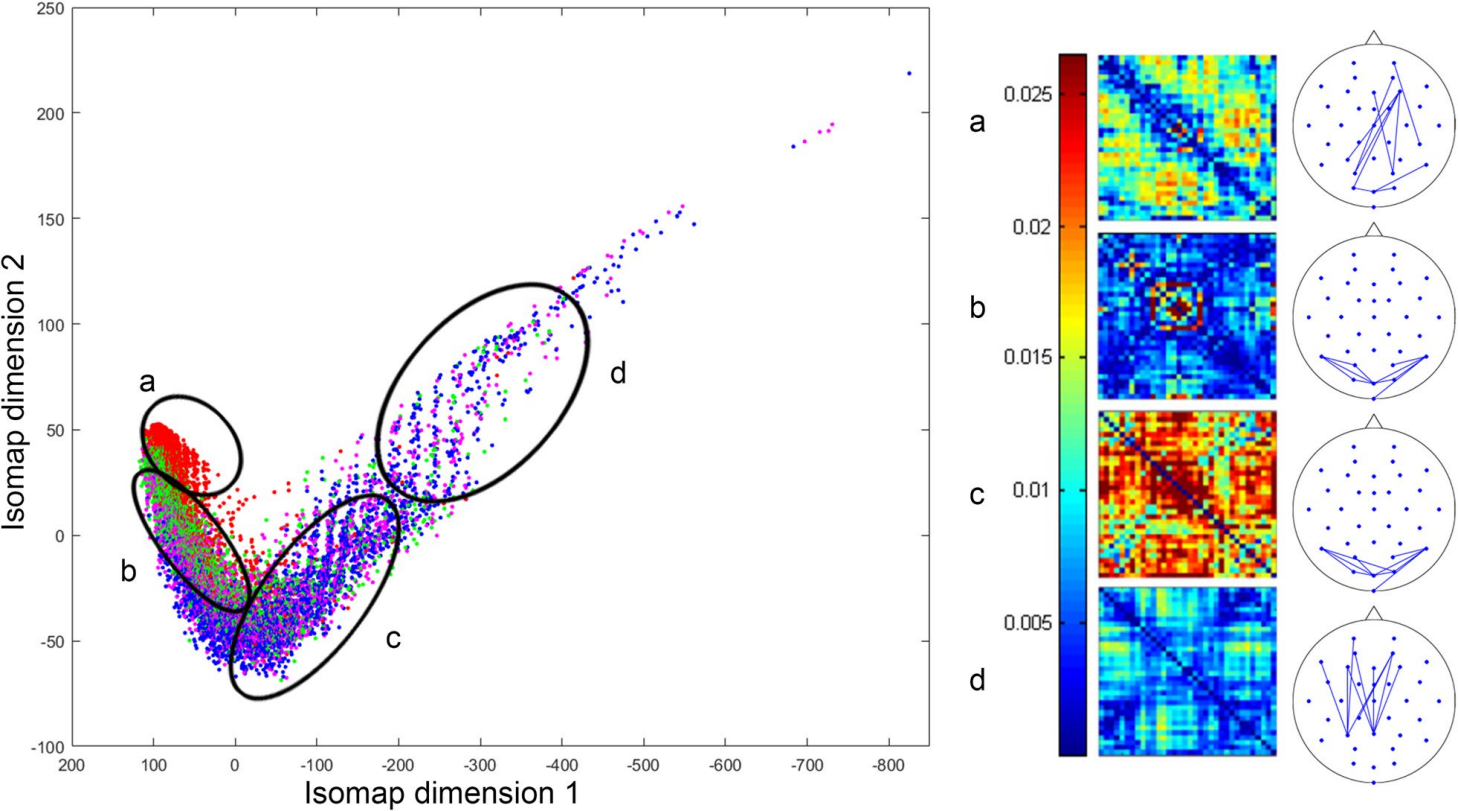
Mean $34\times 34$ theta EEG connectomes of four distinct segments: the head (*a*), the mid and posterior body (*b*, *c*) and the tail (*d*) (left). For each mean connectome, its ten strongest edges were visualized on the layout of the electrodes (right)

**Fig. 5:**
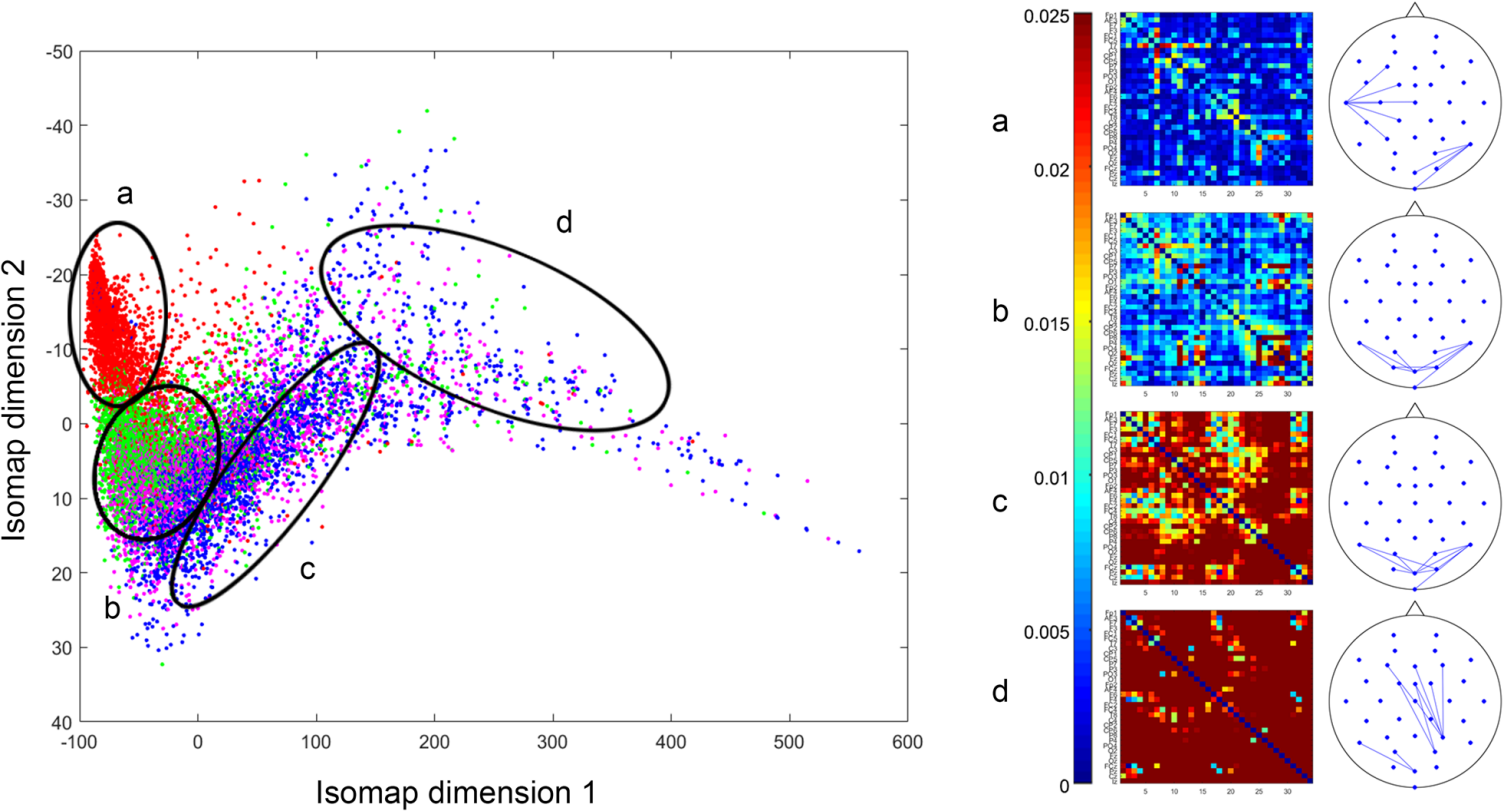
An $\varepsilon$-isomap-based illustration of mean $34\times 34$ theta EEG connectomes of four distinct segments of the manifold: the head (*a*), the mid and posterior body (*b*, *c*) and the tail (*d*) (left). For each mean connectome, its ten strongest edges were visualized on the layout of the electrodes (right)

**Fig. 6:**
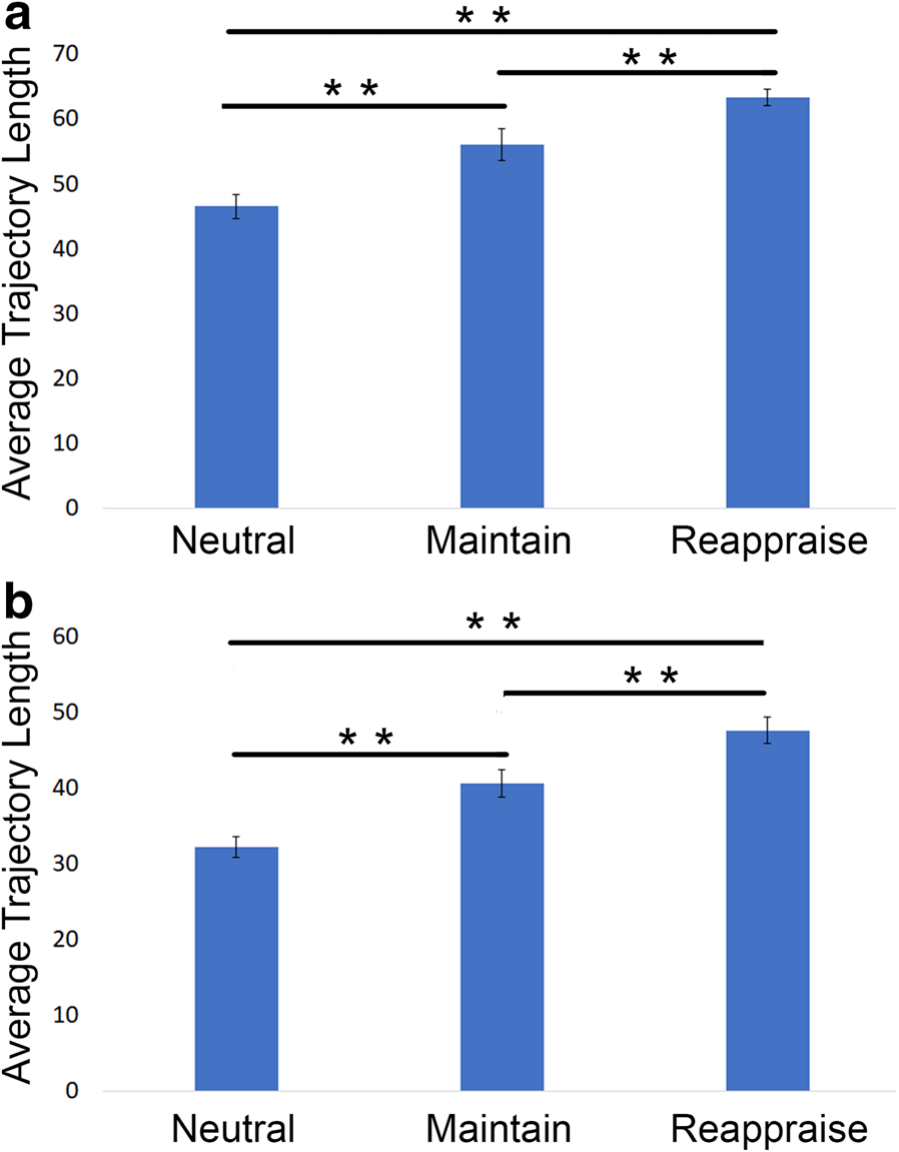
Average trajectory length of each condition in 2-D isomap spaces, both k-nearest neighbor approach (**a**) and the epsilon-based approach (**b**). In k-isomap, the average length in *Neutral*: $46.49 \pm 1.85$, *Maintain*: $55.99 \pm 2.42$, *Reappraise*: $63.30 \pm 1.26$; in $\varepsilon$ -isomap , the average length in *Neutral*: $32.21 \pm 1.37$, *Maintain*: $40.62 \pm 1.82$, *Reappraise*: $47.61 \pm 1.74$. Both suggest that *Thought Chart* travels in longer distance in more complex task conditions. Significant findings in group *t* test are indicated with ^**^$(p<0.01)$ and ^*^$p<0.05$

**Fig. 7:**
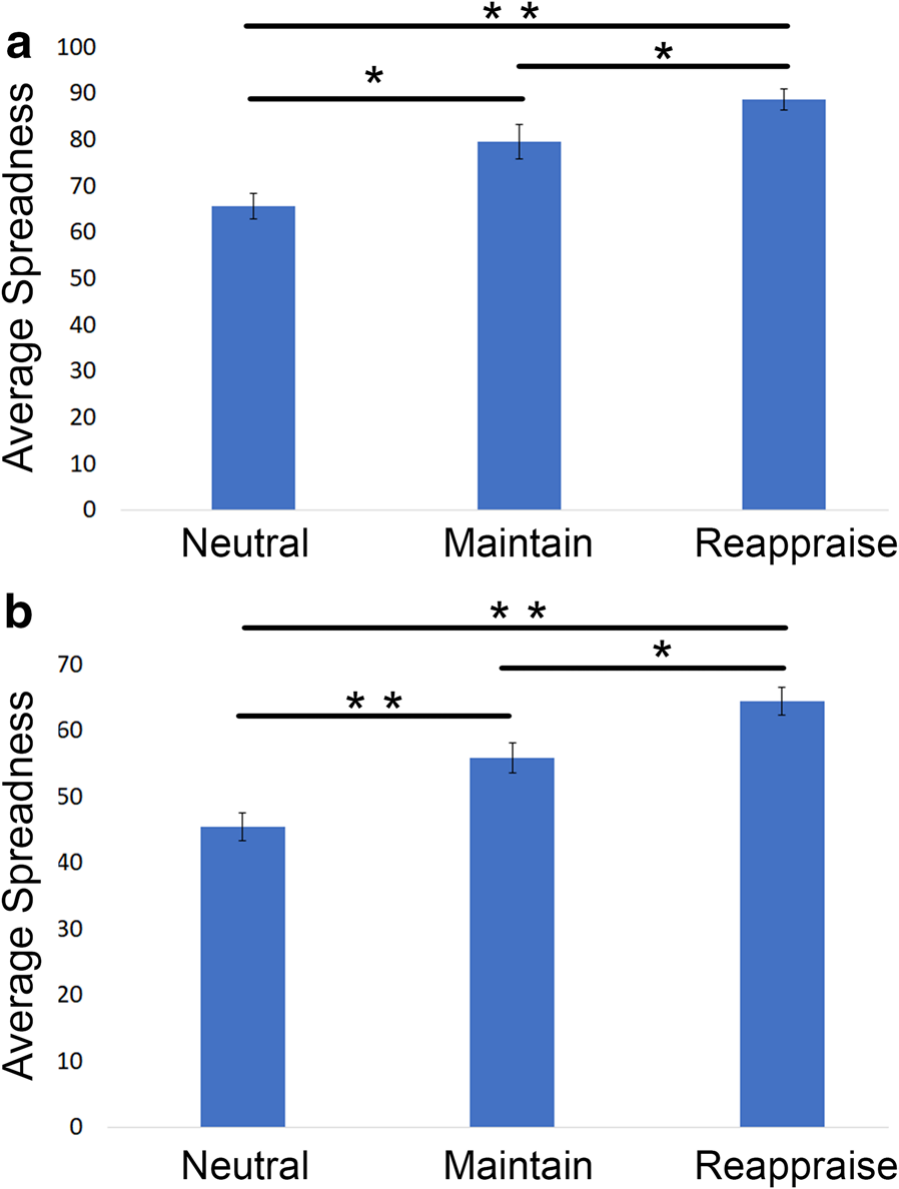
Spreadness of each condition in 2-D isomap spaces, both k-nearest neighbor approach (**a**) and the epsilon-based approach (**b**). In k-isomap, the spreadness in *Neutral*: $65.61\pm 2.74$, *Maintain*: $79.51\pm 3.64$, *Reappraise*: $88.67\pm 2.27$; in $\varepsilon$-isomap, the spreadness in *Neutral*: $45.43\pm 2.09$, *Maintain*: $55.78\pm 2.27$, *Reappraise*: $64.30\pm 2.09$. Both suggest the *Thought Chart* is more scattered as the task condition becomes more complex. Significant findings in group *t* test are indicated with ^**^$p < 0.01$ and ^*^$p < 0.05$

**Fig. 8:**
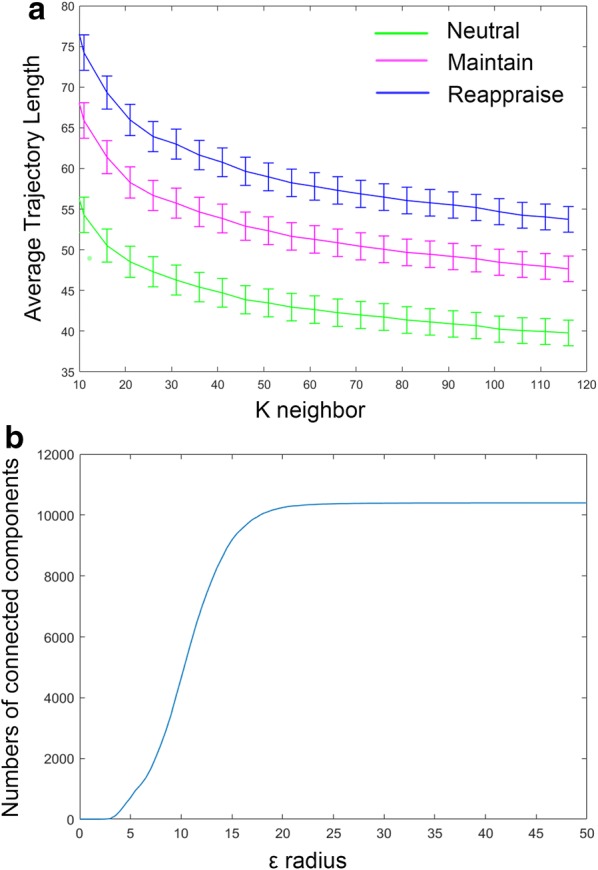
Average trajectory length of *Neutral*, *Maintain* and *Reappraise* across a range of *k* values in the k-nearest neighbor step (**a**) and the number of connected connectomes with a range of $\varepsilon$ values (**b**)

### K-isomap

To further understand theta EEG connectome dynamics, we additionally studied the four distinct subregions of the manifold (i.e., segments of the “snake”):* the head * (primarily resting), *the mid body* (primarily *Neutral*), *the posterior body* (a mixture of *Neutral*, *Maintain*, and *Reappraise*) and *the tail* (primarily *Maintain* and *Reappraise*; Fig. 4). Sampling these segments reveals marked connectome differences. Analysis of the top ten edge strengths in the head region (Fig. 4a) demonstrated increased theta coupling in fronto-parieto-occipital leads while the body (*Neutral*-predominant, Fig. 4b; *Maintain*/*Reappraise* dominant, Fig. 4c) is characterized by predominant theta coupling between occipital leads. Last, the tail (*Maintain*/*Reappraise* only, Fig. 4d) revealed increased theta coupling between frontal and parietal leads.

### *ε*-isomap

*Thought Chart *of the $\varepsilon$-isomap presents a similar distribution along the principle dimension. Taking samples for the head, mid body, posterior body, and tail, the overall connectivity of the average connectome is increasing monotonically, which is different from k-isomap. The top ten edge strengths in the *head* region (Fig. 5a) demonstrated strong parieto-central and occipital theta coupling, while *the body* (Fig. 5b, c) presented increasing theta coupling within occipital channels. Lastly, the increased theta coupling between frontal and occipital leads can be observed at *the tail* (Fig. 5d).

### Average trajectory length and spreadness

In k-isomap *Thought Chart*, average trajectory length in the Euclidean space was significantly longer in more complex tasks (Fig. 6a), (one-way ANOVA: $df=2, F=19.60, \,p<0.001$, paired *t* test: *Neutral* vs. *Maintain*: $p=0.008$, *Maintain* vs. *Reappraise*: $p=0.004$, *Neutral* vs. *Reappraise* $p<0.001$). In $\varepsilon$-isomap *Thought Chart*, a similar trend is observed, where subjects tend to “travel” longer distance in *Thought Chart* during tasks with higher cognitive demand (Fig. 6b). (One-way ANOVA: $df=2,\, F=21.75, \, p<0.001$, paired *t* test: *Neutral* vs. *Maintain*: $p=0.002$, *Maintain* vs. *Reappraise*: $p=0.003$, *Neutral* vs. *Reappraise* $p<0.001$.) Furthermore, task differences not only were reflected in the average distance traveled, but also observed in the level of scattering measured using spreadness (Fig. 7), in both k-isomap (one-way ANOVA: $df=2, F=15.61 \,p<0.001,$ paired *t* test: *Neutral* vs. *Maintain*: $p=0.015$, *Maintain* vs. *Reappraise*: $p=0.047$, *Neutral* vs. *Reappraise* $p<0.001$ ) and $\varepsilon$-isomap (one-way ANOVA: $df=2, F=19.20,p<0.001$, paired *t* test: *Neutral* vs. Maintain: $p=0.008$, Maintain vs. *Reappraise*: $p=0.017$, *Neutral* vs. *Reappraise* $p<0.001$.)

## Discussion

In this study, we proposed a novel unsupervised manifold learning framework to construct a state space, in the form of a manifold embedded in 2-D that quasi-isometrically visualizes EEG connectome dynamics. Moreover, in this space one can visualize time-dependent brain activities as a trajectory or *Thought Chart*. Using the temporal dynamic EEG connectome, two neighborhood construction algorithms are applied to visualize the state space in 2-D. Both k-isomap and $\varepsilon$-isomap are able to compose a highly dynamic and complex geometry, where distinct subregions are distributed along the principle dimension (x-axis in the 2-D space). The baseline resting state is concentrated on one end, followed by mostly *Neutral* points that can be observed later along the x-axis, while *Maintain* and *Reappraise* are mixed, from the posterior section on to the end. Note that this transition corresponds to different mental states, suggesting that the manifold has a principal dimension that is primarily linear, and a vertical motion around this principal dimension whose amplitude increases with cognitive demands. In this context, this manifold may resemble dynamical systems on a *torus* [22] (the surface of a doughnut), in that trajectories are generated by the product of distribution along the principal dimension and a *minor* up–down motion in the second dimension.

By sectioning the region where different tasks concentrated, matrices can be defined to represent these regions. As similar *Thought Chart* trajectories are, regional matrices of k-isomap and $\varepsilon$-isomap however can be different. There are few possible explanations. First, even if each region appears to have a similar composition of tasks, due to the different neighborhood constructing strategy, the points being included in the same regions are not necessarily the same. Second, the sectioning of the head, body, and tail is arbitrary; hence, it can only provide an approximate matrix representation without rigorously controlling the number of points of this region. However, the strongest theta couplings in each region are similar between two methods. Based on the strongest coupling, the manifold comprises subspaces representing resting, visual processing (a common feature of *Neutral*, *Maintain*, and *Reappraise*), and cognitive control (a distinct feature of *Reappraise*). Edge strength analyses of the manifold-sampled EEG connectomes demonstrated increased patterns of theta coupling that are highly consistent with previous reports of frequency-band coupling associated with the resting state [23], visual processing [24], and cognitive control [25].

As it is shown in Figs. 2 and 3, *Thought Chart* is able to visualize an individual’s “mind travels” in a two-dimensional space in this 7-s session. It thus allows us to quantify the temporal dynamics of EEG brain networks by looking into properties such as the distribution and trajectory length. For healthy participants, the mind is more or less “static” when the task is simple and becomes more dynamic as the task requires higher cognitive demand. These properties can be extracted as biomarkers for mind state predictions in future applications.

Limitations of our approach merit further discussion. First, the parameter setting for $\varepsilon$-isomap can be very sensitive since $\varepsilon$ is defined as a distance which can be tuned to go either fine (local) or coarse (global) (Fig. 8b). In this dataset, the reconstructed trajectory is stable as the $\varepsilon$ changed within a certain range. However, in future applications, it may create further complexity in determining the optimal $\varepsilon$, especially if the data are sensitive toward $\varepsilon$. Secondly, it may be inappropriate to compare these thought chart metrics in the resting state with those from *Neutral, Maintain*, and *Reappraise*. This is due to the nature of a task-free design (resting state) versus a task-related design during ERT. Unlike in the resting state when research participants were simply instructed to do nothing (and thus leading to mind wandering), in ERT there is a clear “begin” and “end” cue for all *Neutral, Maintain*, and *Reappraise* sessions and participants were instructed to accomplish a certain task between the cues. Thus, we are able to precisely calculate trajectories by averaging across trials and compare them across three task conditions. However, the resting-state data were collected from a continuous session, during which participants were not instructed to think in any particular way. Therefore, the resting-state data were only included as “baseline reference” in dissimilarity embedding and in isomap visualization but not during subsequent quantitative analyses. Moreover, as a quasi-isometric technique isomap aims to preserve the pairwise geodesics on the manifold, i.e., approximating global isometry when the embedding is constrained to a given dimension. By contrast, other classes of local NDR methods such as LLE unfold the manifold by preserving local linear reconstruction relationship (i.e., local parameterization) of each point within its neighborhood. Furthermore, as the *Theorema Egregium* only guaranteed the invariance of Gauss curvature for complete isometric embeddings of two manifolds, it is unclear whether the manifold constructed using one NDR technique is necessarily more “correct.” Nevertheless, both LLE and isomap recover a principal dimension and a up–down motion around it, while simple linear techniques such as PCA did not. We thus posit that the highly structured complex geometry recovered using our framework may indeed inform the hidden properties of brain dynamics and the underlying neurophysiological mechanisms that generate them.
